# Magnifying Endoscopy with Narrow-Band Imaging for Duodenal Neuroendocrine Tumors

**DOI:** 10.3390/jcm12093106

**Published:** 2023-04-24

**Authors:** Gwang Ha Kim, Kiyoun Yi, Dong Chan Joo, Moon Won Lee, Hye Kyung Jeon, Bong Eun Lee

**Affiliations:** 1 Department of Internal Medicine, Pusan National University School of Medicine, Busan 49241, Republic of Korea; yikiyoun@hanmail.net (K.Y.); oceanose@korea.ac.kr (D.C.J.); neofaceoff@hanmail.net (M.W.L.); kyung3842@hanmail.net (H.K.J.);; 2 Biomedical Research Institute, Pusan National University Hospital, Busan 49241, Republic of Korea

**Keywords:** duodenum, magnifying endoscopy, narrow-band imaging, neuroendocrine tumor, subepithelial lesion

## Abstract

Duodenal neuroendocrine tumors (NETs) are rare subepithelial tumors that arise from the neuroendocrine cells beneath the epithelial layer. However, an accurate histopathological diagnosis is difficult when tissue samples are obtained using conventional endoscopic forceps biopsy alone. This study aimed to evaluate the magnifying endoscopy with narrow-band imaging (ME-NBI) findings of duodenal NETs. We retrospectively analyzed a database of 22 duodenal NETs from 21 patients who underwent ME-NBI between January 2011 and June 2022. The ME-NBI, endosonographic, and histopathologic findings of duodenal NETs were analyzed. Nineteen lesions were located in the bulb, two were located in the superior duodenal angle, and one was located in the second portion of the duodenum. Eighteen lesions (82%) had IIa morphology, and nine (41%) had central depression on the surface. On endoscopic ultrasonography, almost all lesions (20/22, 91%) were located in the second and/or third layers, and the median tumor size was 6 mm. During ME-NBI, the microsurface pattern was regular in 18 lesions (82%) and absent in 4 (18%). The microvascular pattern was regular in 17 lesions (77%), irregular in 4 (18%), and absent in 1 (5%). Thickened subepithelial vessels were observed in 15 (68%) lesions. There was no difference in tumor size according to the presence or absence of thickened subepithelial vessels (6.1 ± 1.8 mm vs. 5.9 ± 3.8 mm, *p* = 0.860). In conclusion, the characteristic ME-NBI findings of duodenal NETs were regular microsurface and microvascular patterns and the presence of thickened subepithelial vessels. These ME-NBI features may be useful for differentiating duodenal NETs from other duodenal subepithelial lesions.

## 1. Introduction

Neuroendocrine tumors (NETs) are rare neoplasms that arise from neuroendocrine cells beneath the epithelial layer. Most NETs arise in the gastrointestinal (GI) tract [1]. Duodenal NETs occur less frequently than gastric and rectal NETs, accounting for 2–4% of all NETs [2]. Most duodenal NETs are small, well differentiated, non-functional, located in the proximal duodenum, and rarely present with local or distant metastases at diagnosis [2,3]. Recently, with the widespread use of upper GI endoscopic screening and the development of high-resolution endoscopy, duodenal NETs are being more commonly diagnosed. In particular, since duodenal NETs tend to arise in the deep mucosa and then spread to the submucosa even during the early stages of the disease, these tumors are incidentally found as subepithelial lesions (SELs) covered by the normal duodenal mucosa during screening endoscopy. Thus, an accurate histopathological diagnosis is challenging for duodenal NETs, which are totally covered by normal duodenal mucosa when tissue samples are obtained using conventional endoscopic forceps biopsy alone. A digging biopsy technique (bite-on-bite technique) can be used for the adequate tissue sampling of GI SELs, but this needs at least 4–10 biopsies [4]. In addition, the bleeding risk is increased to 2.8% and the diagnosis yield is unsatisfactory (17–42%) [4,5].

Endoscopic ultrasonography (EUS) is a useful diagnostic modality for duodenal SELs [4]. On EUS, duodenal NETs appear as homogenously hypoechoic lesions with distinct borders in the second and/or third layers of the duodenum. However, these EUS features are also observed in other benign SELs, such as Brunner’s gland hyperplasia or heterotopic pancreas; they are not specific to duodenal NETs [6].

Magnifying endoscopy with narrow-band imaging (ME-NBI) is another useful diagnostic modality for visualizing microsurface (MS) and microvascular (MV) patterns within the superficial layer of the GI mucosa [7]. ME-NBI findings of gastric NETs include a loss of pit structure at the lesion surface and the presence of abnormal, thickened blood vessels beneath the subepithelial capillary network [8,9]. However, there is a paucity of data on the ME-NBI findings of duodenal NETs. Therefore, this study aimed to elucidate the ME-NBI findings of histopathologically confirmed duodenal NETs.

## 2. Materials and Methods

### 2.1. Study Population

Between January 2011 and June 2022, 24 patients with duodenal NETs underwent both ME-NBI and EUS. All lesions were histopathologically confirmed by endoscopic biopsy or resection. Of these, three patients were excluded from our study because of the absence of final histopathological results due to follow-up loss. Consequently, 22 duodenal NETs from 21 patients were included in this study. Patient demographics, imaging results (EUS and ME-NBI), and histopathology were retrospectively analyzed. This study was reviewed and approved by the Institutional Review Board of the Pusan National University Hospital (IRB number: 2211-020-121).

### 2.2. ME-NBI

**Figure 1:**
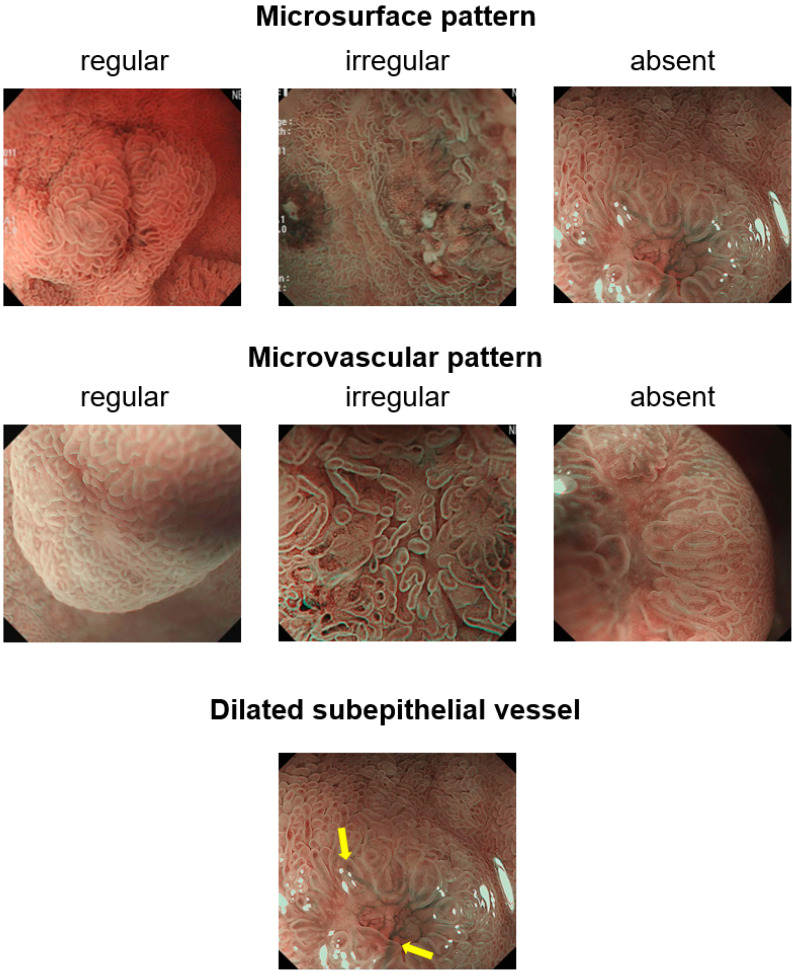
Classification of microsurface and microvascular patterns using magnifying endoscopy with narrow-band imaging in duodenal neuroendocrine tumors.

The EVIS-LUCERA SPECTRUM system (CV-260, CV-290; Olympus, Tokyo, Japan) and magnifying video endoscope (GIF-H260Z, GIF-H290Z; Olympus) were used as video endoscopy systems. For obtaining a clear ME-NBI view, a soft hood (MB-46; Olympus) was attached to the distal tip of the endoscope to the proper focus distance. ME-NBI was performed by a single experienced endoscopist (G.H.K.), and all examinations were performed under conscious sedation with 2–5 mg midazolam. During endoscopy, the following endoscopic features were prospectively recorded for all lesions: (a) location; (b) macroscopic shape (Isp [subpedunculated], Is [sessile], IIa [slightly elevated]), according to the Paris classification of digestive tract lesions [10]; and (c) presence of central depression or ulceration. Subsequently, ME-NBI was performed to evaluate the MS and MV patterns and the presence of abnormally thickened subepithelial vessels (Figure 1).

### 2.3. EUS

EUS was performed using a 20 MHz radial scanning mini-probe (UM3D-DP20-25R; Olympus). After filling the duodenum with 30–100 mL of de-aerated water, the scanning of the lesion was performed. The following EUS features were recorded for all lesions: (a) size; (b) growth pattern (intraluminal, intramural, or extraluminal); (c) sonographic layer of origin; (d) echogenicity (hypoechoic or hyperechoic); (e) homogeneity (homogenous or heterogeneous); and (f) border distinction (distinct or indistinct).

### 2.4. Endoscopic Resection

Endoscopic resections were performed with a conventional single-channel endoscope (GIF-Q260, GIF-H260, GIF-H290; Olympus) under intravenous conscious sedation, as stated previously [11]. For endoscopic mucosal resection (EMR), a mixed solution of normal saline, epinephrine (0.025 mg/mL), and indigo carmine dye was injected into the submucosal layer and the lesion was then resected using a snare. During EMR with a ligation device (EMR-L), the lesion was aspirated into the ligation cap (Stiegmann-Goff ClearVue, ConMed, Boston, MA, USA) after injection of the mixed solution, followed by the deployment of an elastic band. Then, snare resection was performed below the elastic band. For EMR after circumferential precutting (EMR-P), an endoscopic knife (Dual-Knife^®^, Olympus) was used to make markings 2 mm outside the lesion margin. After submucosal injection around the lesion, circumferential incision was performed using the dual knife. Snare resection was then performed with an additional injection beneath the lesion.

### 2.5. Histopathological Evaluation

The resected specimens were fixed in formalin and sectioned serially into 2 mm slices. The tumor size, depth of invasion, tumor grade, presence of lymphovascular invasion, and resection margins were evaluated microscopically. Immunohistochemical staining with synaptophysin, chromogranin A, and CD56 was performed, and the neuroendocrine differentiation and mitotic rate of the tumor were evaluated. Ki-67 staining was also performed to evaluate the tumor cell proliferative activity. The tumors were classified as G1, G2, or G3 based on the mitotic rate (mitoses/2 mm^2^) and Ki-67 index according to the 2019 World Health Organization (WHO) classification [12].

### 2.6. Statistical Analysis

Variables are expressed as medians, ranges, and proportions. The statistical significance of differences in tumor size, according to the presence or absence of thickened subepithelial vessels, was assessed using an independent *t*-test. A *p*-value < 0.05 was considered statistically significant. Statistical calculations were performed using IBM SPSS version 27.0 for Windows (IBM Co., Armonk, NY, USA).

## 3. Results

A total of 22 duodenal NETs in 21 patients (13 males and 8 females; median age, 67 years) were included in the study. Twenty patients had a single lesion and one had two lesions. Nineteen lesions were located in the bulb, two in the superior duodenal angle, and one in the second portion of the duodenum (Table 1). Macroscopically, 18 lesions (82%) had IIa morphology, and 9 (41%) had a central depression on the surface. Endoscopic forceps biopsies were performed in 21 lesions; of them, 17 lesions were confirmed as NETs. The remaining five lesions were confirmed as NETs after endoscopic resection. EMR-L was performed in 20 lesions, EMR was performed in 1, and EMR-P was performed in 1, according to the endoscopist’s preference. Histopathologically, 1 tumor was confined to the mucosa and 21 extended to the submucosa. The grading, according to the WHO classification, showed G1 for 19 tumors and G2 for 3.

On EUS, almost all lesions (20/22, 91%) were located in the second (deep mucosal) and third (submucosal) layers, with a median size of 6 mm (range 2–11 mm) (Table 2). All lesions showed an intramural growth pattern, a homogeneous hypoechoic echogenicity, and distinct borders.

During ME-NBI, a regular MS pattern was observed in 18 lesions (82%), and an absent MS pattern was observed in 4 lesions (18%) (Table 3). The MV pattern was regular in 17 lesions (77%), irregular in 4 lesions (18%), and absent in 1 lesion (5%). Abnormally thickened subepithelial vessels were observed in 15 lesions (68%). Thickened subepithelial vessels were not observed in one case, in which the tumor was confined to the mucosa only, but were observed in 15 of the 21 cases (71%), in which the tumor extended to the submucosa. When we evaluated the tumor size according to the presence or absence of thickened subepithelial vessels, there was no association (6.1 ± 1.8 mm vs. 5.9 ± 3.8 mm, *p* = 0.860).

## 4. Discussion

Duodenal NETs appear as firm, round, or oval SELs in conventional endoscopy. While central surface depression is an endoscopic finding that is suggestive of duodenal NETs, it is present in less than half of cases [13]. Central depression is also observed in other SELs, such as ectopic pancreas or Brunner’s gland hyperplasia. Furthermore, a histopathological diagnosis is not usually possible using endoscopic forceps biopsy. Characteristic EUS features, such as homogeneous hypoechogenicity, distinct borders, and location within the second and/or third layers, are helpful for diagnosing NETs but are non-specific [6]. Therefore, we used ME-NBI to identify additional findings to predict duodenal NETs. We demonstrated that abnormally thickened subepithelial vessels were present in more than two-thirds of lesions. To our knowledge, this study is the first to report ME-NBI findings of duodenal NETs.

ME-NBI is a useful diagnostic modality with a maximal resolution power of 6.4 µm for visualizing real-time microscopic images of the mucosal surface [14,15]. The clinical utility of ME-NBI has been investigated mainly in relation to GI epithelial neoplasms [7,16]; however, reports on the use of ME-NBI in GI SELs are rare [17]. Histopathologically, NETs arise from neuroendocrine cells, which are mainly present in the deep mucosa and tend to grow into the submucosa, resulting in the covering of the normal epithelium. In the present study, approximately 80% of duodenal NETs showed normal MS and/or MV patterns. When the tumor cells grow into the epithelium, the thinning or disruption of the epithelium can occur, leading to epithelial loss or the exposure of subepithelial tumor microvessels. In the present study, four lesions (18%) showed an absent MS pattern and four (18%) showed an irregular MV pattern. The frequencies of normal MS and MV patterns in duodenal NETs (82% and 77%) were higher than those in polypoid type I gastric NETs (40% and 53%) evaluated using NBI alone [18]. This difference could be explained by epithelial injury above NETs caused by gastric acid and the morphological difference of the NETs included in the study.

When the tumor grows expansively beneath the epithelium, a greater blood supply is needed for tumor cell growth within the deep mucosa and submucosa. Accordingly, new subepithelial vessels or the dilation of normal subepithelial vessels may appear. Several case reports have demonstrated the presence of abnormally dilated subepithelial vessels in various GI NETs [8,9,19,20]. During ME-NBI, abnormally thickened subepithelial vessels were observed in 15 lesions (68%). These vessels were not observed in one case limited to the mucosa but were observed in 15 cases (71%) with submucosal extension. However, there was no difference in tumor size based on the presence or absence of thickened subepithelial vessels.

It is unclear whether thickened subepithelial vessels are only observed in NETs. We have previously reported thickened subepithelial vessels in a heterotopic pancreas of the stomach [17]. Thickened subepithelial vessels are likely an indirect sign of subepithelial components within the deep mucosa or submucosa, such as endocrine nests in NETs or pancreatic acinar nests in heterotopic pancreas [17,19,21]. To date, there is a lack of data on ME-NBI findings for duodenal SELs. Thus, further studies involving a larger number of patients with duodenal SELs are warranted.

This study had several limitations. First, this was a single-center, retrospective study that assessed the ME-NBI findings of duodenal NETs. Therefore, there was a risk of selection and potential bias when selecting patients and retrospectively reviewing ME-NBI findings. Second, ME-NBI was performed by a single experienced endoscopist and interobserver variation was not evaluated. Finally, the incidence of small bowel NETs in the United States is reported to be 1.05 per 100,000 inhabitants [22]; therefore, the number of included cases was small owing to the rarity of duodenal NETs, and more aggressive tumors, such as G3, were not included. Therefore, a multicenter study involving a larger number of duodenal NETs is needed. Despite these limitations, this study is valuable for evaluating ME-NBI findings predictive of duodenal NETs.

In conclusion, the characteristic ME-NBI findings of duodenal NETs include regular MS/MV patterns and the presence of thickened subepithelial vessels. These ME-NBI features are potentially useful for differentiating NETs from other duodenal SELs.

## Figures and Tables

**Table 1:** Summary of conventional endoscopy, ME-NBI, and EUS findings in 21 patients with duodenal neuroendocrine tumors.

Case	Sex	Age (Years)	Conventional Endoscopy			ME-NBI			EUS					
			Location	Macroscopic Shape	Central Depression	MS Pattern	MV Pattern	Thickened Subepithelial Vessels	Size (mm)	Growth Pattern	Layer	Echogenicity	Homogeneity	Border
1	M	53	Bulb	IIa	Absent	Regular	Regular	Absent	3	Intramural	2	Hypoechoic	Homogenous	Distinct
2	M	75	SDA	IIa	Present	Regular	Regular	Present	2	Intramural	2, 3	Hypoechoic	Homogenous	Distinct
3	M	64	SDA	Isp	Absent	Regular	Regular	Present	8	Intramural	2, 3	Hypoechoic	Homogenous	Distinct
4	F	79	Bulb	IIa	Absent	Absent	Irregular	Present	6	Intramural	2, 3	Hypoechoic	Homogenous	Distinct
5	F	79	Bulb	IIa	Absent	Absent	Irregular	Present	6	Intramural	2, 3	Hypoechoic	Homogenous	Distinct
			Bulb	IIa	Absent	Regular	Regular	Present	6	Intramural	2, 3	Hypoechoic	Homogenous	Distinct
6	M	38	Bulb	IIa	Present	Regular	Regular	Present	4	Intramural	2, 3	Hypoechoic	Homogenous	Distinct
7	M	69	Bulb	IIa	Present	Regular	Regular	Present	6	Intramural	2, 3	Hypoechoic	Homogenous	Distinct
8	F	68	Bulb	IIa	Present	Regular	Regular	Present	8	Intramural	2, 3	Hypoechoic	Homogenous	Distinct
9	M	71	Bulb	IIa	Absent	Regular	Irregular	Present	5	Intramural	2, 3	Hypoechoic	Homogenous	Distinct
10	M	74	Bulb	IIa	Absent	Regular	Regular	Absent	2	Intramural	2, 3	Hypoechoic	Homogenous	Distinct
11	M	40	Second	IIa	Absent	Regular	Regular	Absent	2	Intramural	2, 3	Hypoechoic	Homogenous	Distinct
12	F	82	Bulb	IIa	Absent	Regular	Regular	Present	7	Intramural	2, 3	Hypoechoic	Homogenous	Distinct
13	M	40	Bulb	Is	Absent	Regular	Regular	Absent	5	Intramural	2	Hypoechoic	Homogenous	Distinct
14	M	51	Bulb	Is	Absent	Regular	Regular	Absent	11	Intramural	2, 3	Hypoechoic	Homogenous	Distinct
15	M	67	Bulb	IIa	Present	Regular	Regular	Present	8	Intramural	2, 3	Hypoechoic	Homogenous	Distinct
16	F	43	Bulb	IIa	Present	Absent	Irregular	Present	5	Intramural	2, 3	Hypoechoic	Homogenous	Distinct
17	M	73	Bulb	Is	Present	Absent	Absent	Absent	10	Intramural	2, 3	Hypoechoic	Homogenous	Distinct
18	M	65	Bulb	IIa	Present	Regular	Regular	Present	5	Intramural	2, 3	Hypoechoic	Homogenous	Distinct
19	F	74	Bulb	IIa	Absent	Regular	Regular	Absent	8	Intramural	2, 3	Hypoechoic	Homogenous	Distinct
20	F	50	Bulb	IIa	Present	Regular	Regular	Present	9	Intramural	2, 3	Hypoechoic	Homogenous	Distinct
21	F	48	Bulb	IIa	Absent	Regular	Regular	Present	7	Intramural	2, 3	Hypoechoic	Homogenous	Distinct

ME-NBI, magnifying endoscopy with narrow-band imaging; EUS, endoscopic ultrasonography; MS, microsurface; MV, microvascular; SDA, superior duodenal angle.

**Table 2:** Demographic, endoscopic, and endosonographic characteristics of 21 patients (22 lesions) with duodenal neuroendocrine tumors.

***Patient characteristics***	
Median age (years, range)	67 (40–82)
Sex, *n* (%)	
Male	13 (62)
Female	8 (38)
***Conventional endoscopic characteristics***	
Location, *n* (%)	
Bulb	19 (86)
Superior duodenal angle	2 (9)
Second portion	1 (5)
Macroscopic shape, *n* (%)	
Isp	1 (5)
Is	3 (14)
IIa	18 (82)
Central depression, *n* (%)	
Present	9 (41)
Absent	13 (59)
***Endosonographic characteristics***	
Median size (mm, range)	6 (2–11)
Growth pattern, *n* (%)	
Intramural	22 (100)
Layer, *n* (%)	
Second layer	2 (9)
Second and third layer	20 (91)
Echogenicity, *n* (%)	
Hypoechoic	22 (100)
Homogeneity, *n* (%)	
Homogenous	22 (100)
Border, *n* (%)	
Distinct	22 (100)

**Table 3:** Magnifying endoscopy with narrow-band imaging of duodenal neuroendocrine tumors.

Microsurface pattern, *n* (%)	
Regular	18 (82)
Irregular	0 (0)
Absent	4 (18)
Microvascular pattern, *n* (%)	
Regular	17 (77)
Irregular	4 (18)
Absent	1 (5)
Thickened submucosal vessel, *n* (%)	
Present	15 (68)
Absent	7 (32)

## Data Availability

The data presented in this study are available on request from the corresponding author.

## Abbreviations

EMR: endoscopic mucosal resection; EMR-L: EMR with a ligation device; EMR-P: EMR after circumferential precutting; EUS: endoscopic ultrasonography; GI: gastrointestinal; ME-NBI: magnifying endoscopy with narrow-band imaging; MS: microsurface; MV: microvascular; NET: neuroendocrine tumor; SEL: subepithelial lesion; WHO: World Health Organization.

## References

[1] Modlin I.M., Sandor A. (1997). An analysis of 8305 cases of carcinoid tumors. Cancer.

[2] Yao J.C., Hassan M., Phan A., Dagohoy C., Leary C., Mares J.E., Abdalla E.K., Fleming J.B., Vauthey J.N., Rashid A. (2008). One hundred years after ”carcinoid”: Epidemiology of and prognostic factors for neuroendocrine tumors in 35,825 cases in the United States. J. Clin. Oncol..

[3] Hoffmann K.M., Furukawa M., Jensen R.T. (2005). Duodenal neuroendocrine tumors: Classification, functional syndromes, diagnosis and medical treatment. Best. Pract. Res. Clin. Gastroenterol..

[4] Kim G.H. (2022). Systematic endoscopic approach for diagnosing gastric subepithelial tumors. Gut Liver.

[5] Ji J.S., Lee B.I., Choi K.Y., Kim B.W., Choi H., Huh M., Chung W.C., Chae H.S., Chung I.S. (2009). Diagnostic yield of tissue sampling using a bite-on-bite technique for incidental subepithelial lesions. Korean J. Intern. Med..

[6] Kim T.W., Kim G.H., Park D.Y., Ahn S., Lim W., Lee B.E., Song G.A. (2017). Endoscopic resection for duodenal subepithelial tumors: A single-center experience. Surg. Endosc..

[7] Lee W. (2021). Application of current image-enhanced endoscopy in gastric diseases. Clin. Endosc..

[8] Singh R., Yao K., Anagnostopoulos G., Kaye P., Ragunath K. (2008). Microcarcinoid tumor diagnosed with high-resolution magnification endoscopy and narrow band imaging. Endoscopy.

[9] Hirai M., Matsumoto K., Ueyama H., Fukushima H., Murakami T., Sasaki H., Nagahara A., Yao T., Watanabe S. (2013). A case of neuroendocrine tumor G1 with unique histopathological growth progress. World J. Gastrointest. Endosc..

[10] Endoscopic Classification Review Group (2005). Update on the Paris classification of superficial neoplastic lesions in the digestive tract. Endoscopy.

[11] Kim G.H., Kim J.I., Jeon S.W., Moon J.S., Chung I.K., Jee S.R., Kim H.U., Seo G.S., Baik G.H., Lee Y.C. (2014). Endoscopic resection for duodenal carcinoid tumors: A multicenter, retrospective study. J. Gastroenterol. Hepatol..

[12] Nagtegaal I.D., Odze R.D., Klimstra D., Paradis V., Rugge M., Schirmacher P., Washington K.M., Carneiro F., Cree I.A., WHO Classification of Tumours Editorial Board (2020). The 2019 WHO classification of tumours of the digestive system. Histopathology.

[13] Yoon J.Y., Kumta N.A., Kim M.K. (2021). The role of endoscopy in small bowel neuroendocrine tumors. Clin. Endosc..

[14] Chuman K., Yao K., Kanemitsu T., Nagahama T., Miyaoka M., Takahashi H., Imamura K., Hasegawa R., Ueki T., Tanabe H. (2021). Histological architecture of gastric epithelial neoplasias that showed absent microsurface patterns, visualized by magnifying endoscopy with narrow-band imaging. Clin. Endosc..

[15] Yao K. (2015). Clinical application of magnifying endoscopy with narrow-band imaging in the stomach. Clin. Endosc..

[16] Kim S.H., Hong S.J. (2021). Current status of image-enhanced endoscopy for early identification of esophageal neoplasms. Clin. Endosc..

[17] Oh H., Kim G.H., Lee M.W., Jeon H.K., Baek D.H., Lee B.E. (2018). Magnifying endoscopy with narrow-band imaging for gastric heterotopic pancreas. Endosc. Int. Open.

[18] Lahner E., Esposito G., Angeletti S., Corleto V.D., Pilozzi E., Di Giulio E., Annibale B. (2016). Endoscopic appearances of polypoid type 1 gastric microcarcinoids by narrow-band imaging: A case series in a referral center. Eur. J. Gastroenterol. Hepatol..

[19] Sato Y. (2015). Endoscopic diagnosis and management of type I neuroendocrine tumors. World J. Gastrointest. Endosc..

[20] Furukawa K., Nakamura M., Kawashima H., Fujishiro M. (2022). Endoscopic resection of a duodenal neuroendocrine tumor. Rev. Esp. Enferm. Dig..

[21] Nojiri T., Ikegami M. (2001). Multiple minute carcinoids in type A gastritis: Attempt at 3-D reconstruction. Pathol. Int..

[22] Dasari A., Shen C., Halperin D., Zhao B., Zhou S., Xu Y., Shih T., Yao J.C. (2017). Trends in the incidence, prevalence, and survival outcomes in patients with neuroendocrine tumors in the United States. JAMA Oncol..

